# STRING v10: protein–protein interaction networks, integrated over the tree of life

**DOI:** 10.1093/nar/gku1003

**Published:** 2014-10-28

**Authors:** Damian Szklarczyk, Andrea Franceschini, Stefan Wyder, Kristoffer Forslund, Davide Heller, Jaime Huerta-Cepas, Milan Simonovic, Alexander Roth, Alberto Santos, Kalliopi P. Tsafou, Michael Kuhn, Peer Bork, Lars J. Jensen, Christian von Mering

**Affiliations:** 1 Institute of Molecular Life Sciences and Swiss Institute of Bioinformatics, University of Zurich, 8057 Zurich, Switzerland; 2 European Molecular Biology Laboratory, 69117 Heidelberg, Germany; 3 Novo Nordisk Foundation Center for Protein Research, University of Copenhagen, 2200 Copenhagen N, Denmark; 4 Biotechnology Center, Technische Universität Dresden, 01062 Dresden, Germany; 5 Max Planck Institute of Molecular Cell Biology and Genetics, 01062 Dresden, Germany

## Abstract

The many functional partnerships and interactions that occur between proteins are at the core of cellular processing and their systematic characterization helps to provide context in molecular systems biology. However, known and predicted interactions are scattered over multiple resources, and the available data exhibit notable differences in terms of quality and completeness. The STRING database (http://string-db.org) aims to provide a critical assessment and integration of protein–protein interactions, including direct (physical) as well as indirect (functional) associations. The new version 10.0 of STRING covers more than 2000 organisms, which has necessitated novel, scalable algorithms for transferring interaction information between organisms. For this purpose, we have introduced hierarchical and self-consistent orthology annotations for all interacting proteins, grouping the proteins into families at various levels of phylogenetic resolution. Further improvements in version 10.0 include a completely redesigned prediction pipeline for inferring protein–protein associations from co-expression data, an API interface for the *R* computing environment and improved statistical analysis for enrichment tests in user-provided networks.

## INTRODUCTION

For a full description of a protein's function, knowledge about its specific interaction partners is an important prerequisite. The concept of protein ‘function’ is somewhat hierarchical (1–4), and at all levels in this hierarchy, interactions between proteins help to describe and narrow down a protein's function: its three-dimensional structure may become meaningful only in the context of a larger protein assembly, its molecular actions may be regulated by co-operative binding or allostery, and its cellular context may be controlled by a multitude of transport, sequestering, and signaling interactions. Given this importance of interactions, many protein annotation and classification schemes assign groups of interacting proteins into functional sets, designated either as physical complexes, signaling pathways or tightly linked ‘modules’ (1,5–7). However, the partitioning of interactions into distinct pathways or complexes can be somewhat arbitrary, and may not do justice to the prevalence of crosstalk and dynamic variation in the interaction landscape (8). A widely used concept that avoids partitioning of function arbitrarily is the *protein network*, i.e. the topological summary of all known or predicted protein interactions in an organism. For functional studies, arguably the most useful networks are those that integrate all types of interactions: stable physical associations, transient binding, substrate chaining, information relay and others. The STRING database (Search Tool for the Retrieval of Interacting Genes/Proteins) is dedicated to such *functional associations* between proteins, on a global scale.

Protein–protein interaction information can already be retrieved from a number of online resources. First, primary interaction databases (e.g. 9–13) which are largely collaborating (14,15) provide curated experimental data originating from a variety of biochemical, biophysical and genetic techniques. Second, since protein–protein interactions can also be predicted computationally, a number of resources have their main focus on interaction prediction, using a variety of algorithms (e.g. 16–20). Lastly, a group of online resources is providing an integration of both known and predicted interactions, thus aiming for high comprehensiveness and coverage. These include STRING, as well as GeneMANIA (21), FunCoup (18), I2D (22), ConsensusPathDB (22) and others. Within this landscape of online resources, STRING places its focus on interaction confidence scoring, comprehensive coverage (in terms of number of proteins, organisms and prediction methods), intuitive user interfaces and on a commitment to maintain a long-term, stable resource (since 2000).

The basic interaction unit in STRING is the *functional association*, i.e. a specific and productive functional relationship between two proteins, likely contributing to a common biological purpose. Interactions are derived from multiple sources: (i) known experimental interactions are imported from primary databases, (ii) pathway knowledge is parsed from manually curated databases, (iii) automated text-mining is applied to uncover statistical and/or semantic links between proteins, based on Medline abstracts and a large collection of full-text articles, (iv) interactions are predicted *de novo* by a number of algorithms using genomic information (23–25) as well as by co-expression analysis and (v) interactions that are observed in one organism are systematically transferred to other organisms, via pre-computed orthology relations. STRING centers on protein-coding gene loci—alternative splice isoforms or post-translationally modified forms are not resolved, but are instead collapsed at the level of the gene locus. All sources of interaction evidence are benchmarked and calibrated against previous knowledge, using the high-level functional groupings provided by the manually curated Kyoto Encyclopedia of Genes and Genomes (KEGG) pathway maps (5).

As of the current update to version 10.0, the number of organisms covered by STRING has increased to 2031, almost doubling over the previous release. The update also encompassed importing and processing all primary data sources again, re-running all prediction algorithms and re-executing the entire text-mining pipeline with new dictionaries and extended text collections. Many of the features and interfaces of STRING have already been described previously (26–28). Below, we have given a short overview of the resource and describe recent additions and modifications.

### User interface

The main entry point into the STRING website is the protein search box on its start page. It supports queries for multiple proteins, can be restricted to certain organisms or clades of organisms, and uses a weighted scheme to rank annotation text matches and identifier matches. Users can also arrive via a number of external websites (29–32) that maintain cross-links with STRING, including the partner resources Search Tool for Interactions of Chemicals (STITCH; 33) and eggNOG (34)—the latter both share protein sequences, annotations and name-spaces with STRING. A third way to enter STRING is via logging on to the *My Data* section; this allows users to upload gene-lists, create identifier mappings, view their browsing history and provide additional ‘payload’ data to be displayed alongside the interactions.

**Figure 1.**
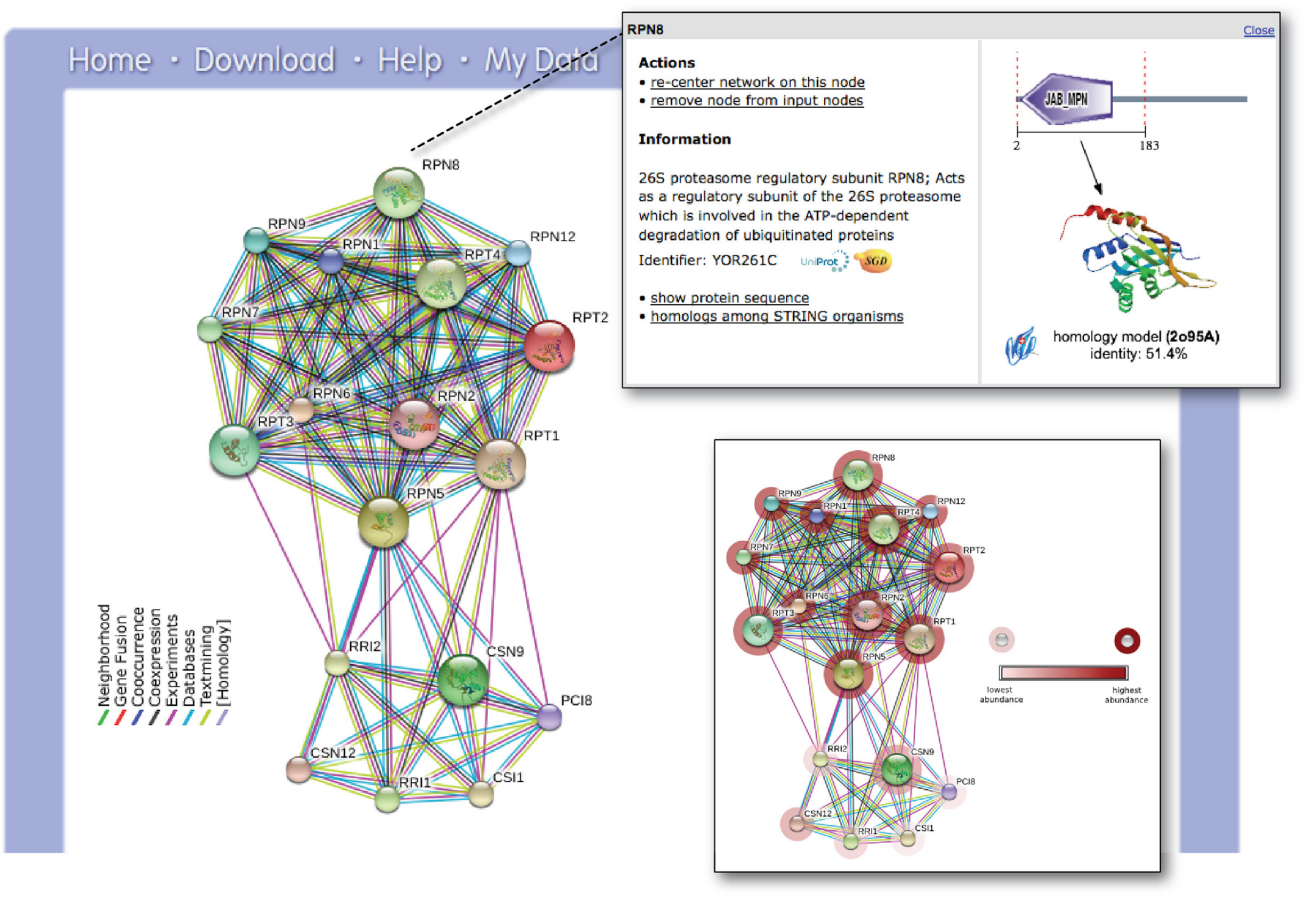
The STRING network view. Combined screenshots from the STRING website, which has been queried with a subset of proteins belonging to two different protein complexes in yeast (the COP9 signalosome, as well as the proteasome). Colored lines between the proteins indicate the various types of interaction evidence. Protein nodes which are enlarged indicate the availability of 3D protein structure information. Inset top right: for each protein, accessory information is available which includes annotations, cross-links and domain structures. Inset bottom right: the same network is shown after the addition of a user-configurable ‘payload’-dataset (26). In this case, the payload corresponds to color-coded protein abundance information, and reveals systematic differences in the expression strength of both complexes.

Once a protein or set of proteins is identified, users proceed to the network view (Figure 1). From there, it is possible to inspect the interaction evidence, to re-adjust the score-cutoffs and network size limits and to view detailed information about the interacting proteins. Upon switching to the ‘advanced’ mode (via the tool panel below the network), users can also cluster and rearrange the network and test for statistical enrichments in the network. The latter feature has been enhanced for the current version 10.0 of STRING: enrichment detection now also covers human disease associations and tissue annotations, which might be statistically enriched in a given network. For this feature, STRING connects with the partner databases TISSUES (http://tissues.jensenlab.org) and DISEASES (http://diseases.jensenlab.org), which also share sequence and name spaces with STRING, and which annotate proteins to tissues or to disease entities based on a combination of automated text-mining and knowledge imports.

### Interaction transfer between organisms

Since version 6.0 of STRING, a significant source of interactions for any given organism has been the transfer of interaction knowledge from orthologous proteins observed to be interacting in another organism. Since version 9.1, these so-called ‘interolog’ transfers were based on pre-computed orthology relations imported from the eggNOG database (34). Orthologs in eggNOG are provided in a hierarchical and nested fashion, allowing the transfer of interactions by traversing up and down along the hierarchy of clades in the tree of life (26). For this purpose, the nested orthology assignments should ideally be fully self-consistent: proteins assigned to an orthologous group for a given phylogenetic clade should be grouped together in all higher-level clades too. In past versions of the orthologous groups, this has not always been the case for technical reasons (orthology assignments are computed independently for each clade). However, for STRING v10, a post-processing pipeline has been devised that makes the orthology setup fully self-consistent. It implements consistency by iteratively splitting and merging orthologous groups at the various clades and levels, until a fully consistent state is achieved. As of now, this post-processed set of orthologs forms the basis for all interaction-transfers in STRING v10. In future releases, the same hierarchical and consistent set of protein families and orthologs will be used also for more intuitive navigation and search features on the user interface.

### Co-expression analysis

**Figure 2.**
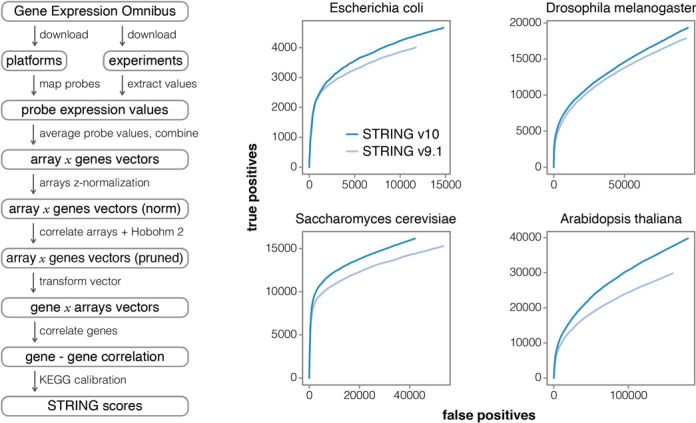
Improved Co-expression analysis. STRING v10 features a completely re-designed pipeline for accessing and processing gene expression information. Left: overview of the individual steps; note that redundant expression experiments are now detected and pruned automatically. Right: improved benchmark performance of the resulting co-expression links, relative to the previous version of STRING, in four model organisms (ROC curves). The benchmark is based on the KEGG pathway maps; predicted interactions are considered to be true positives when both interacting proteins are annotated to the same KEGG map.

It has long been established that co-expression is a proxy for co-regulation (35,36) and a strong indicator of functional associations. The co-expression scores in STRING v10 are computed using a revised and improved pipeline (Figure 2), making use of all microarray gene expression experiments deposited in NCBI Gene Expression Omnibus (NCBI GEO) (37). As of March 2014, GEO consisted of more than 12 000 different platforms (GPL), 45 000 experiments (GSE) and over 1 million matrices (GSM). By including the large amount of diverse arrays in the analysis we can decrease the bias of individual platforms and experiments, and reduce the impact of non-informative matrices. Prior to the analysis, 22 organisms were identified as providing sufficient data (at least 50 experiments each). The first step of the pipeline maps probe identifiers from each platform file (GPL) to STRING genes, using dictionaries from the text-mining pipeline. Samples with less than 100 map-able genes and experiments with less than three samples are excluded from further analysis. The microarray expression values (extracted from the GSE files) are then normalized (*z*-value normalization) and values for each probe merged into single vectors (separately for single-channel and dual-channel arrays). Additionally, single-channel array values are log_2_-transformed and their mean is subtracted, to make them compatible with fold-change values in the two-channel case. Expression values of genes measured by more than one probe are averaged. In order to remove the redundancy and to increase information density between the arrays, the gene expression vectors are correlated with one another (using Spearman's rank correlation) and the full set of arrays is pruned using the Hobohm-2 algorithm (38) with similarity thresholds of 0.7 and 0.95, for single-channel and dual-channel arrays, respectively. The new gene expression values are then correlated gene-by-gene (Pearson correlation) and the resulting values are calibrated against common membership in KEGG pathway maps (release 2014-07-21) in order to compute STRING scores. Lastly, the scores from single- and dual-channel arrays are combined in a probabilistic manner to get the final scores. KEGG benchmark performance clearly improves relative to STRING v9.1 (Figure 2). The improvements can be attributed to the increased size of the GEO repository (experiments added since 2011) and to changes in our pipeline, namely: (i) the additional step to prune highly correlated samples using the Hobohm-2 algorithm and (ii) several minor improvements and bug fixes.

### R/Bioconductor access

**Figure 3.**
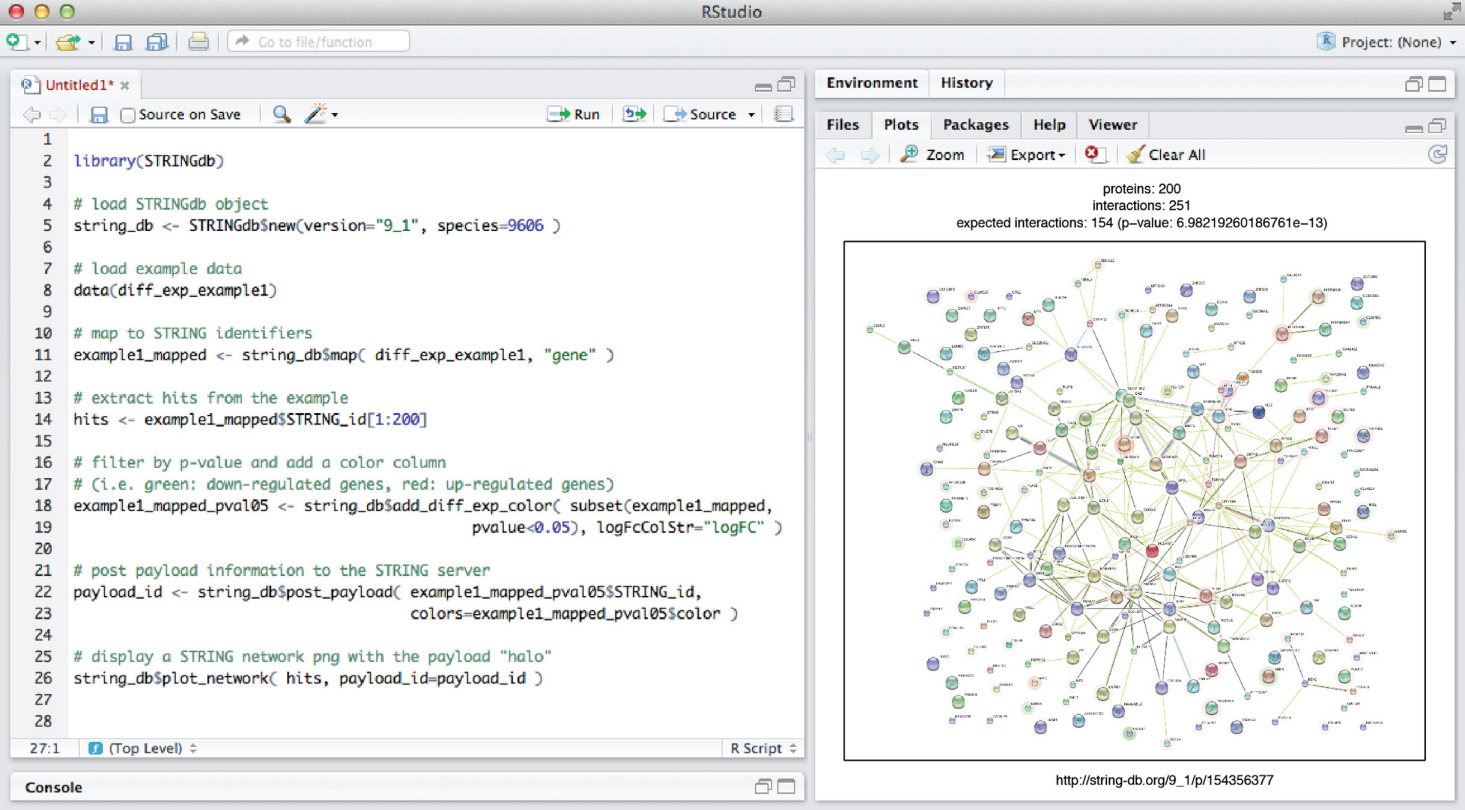
Access to STRING from R/Bioconductor. Left: example session describing how to initialize a human protein network from the STRING database backend, and how to map a set of gene names against it. A subset of the proteins is then plotted as a STRING network (right), complete with auxiliary numerical payload-information highlighting some nodes of interest (red color halos).

Apart from directly browsing and searching the website, data access in STRING is possible also via a REST-based API (application programing interface) and via wholesale data download. With version 10.0, we have introduced a further option: direct access from the *R* programming environment, following the Bioconductor standard (39). The corresponding package is named *STRINGdb* (Figure 3), and can be downloaded from the Bioconductor repository (http://www.bioconductor.org/packages/release/bioc/html/STRINGdb.html). The package interacts with the STRING server via the REST API and via additional, dedicated web services. To optimize the speed of subsequent accesses, the entire interaction network and associated data for a given organism are downloaded from the server and cached locally in the *R* environment, whenever possible. The package is built around the iGraph framework (40), which handles the complexity of the network data structures and provides fast query/analysis functions. Once a network is loaded/cached into an iGraph object, high-level functions facilitate the most common user tasks, such as mapping protein names onto their corresponding STRING identifiers, retrieving the neighbors of a protein of interest, retrieving PubMed IDs for publications that support a given interaction, finding clusters of proteins in the network and generating stable links back to the STRING website.

The *plot_network* function can be used to display a native STRING network of proteins in *R* (Figure 3). Functions are also available to augment a given network with user-provided node colorings (‘payload information’, see also Figure 1), such that subsets of proteins can be tagged and visually highlighted. Statistical enrichment tests can be executed on gene lists within the STRING namespace, covering Gene Ontology and pathway annotations, as well as tissue and diseases annotations. Results can be visualized as lists of enriched terms and/or heatmaps. The R-package proves particularly valuable for users arriving with a very large set of genes, for which the web-based interface of STRING has previously been a major bottleneck.
